# Challenging Management of a Severe Bronchopleural Fistula Caused by a Persistent Air Leak

**DOI:** 10.7759/cureus.90025

**Published:** 2025-08-13

**Authors:** Cameron Bondy, Shaun Sidhu, Spiros Kavvathas, Mufadda Hasan

**Affiliations:** 1 Internal Medicine, Arrowhead Regional Medical Center, Colton, USA; 2 Pulmonary and Critical Care Medicine, Arrowhead Regional Medical Center, Colton, USA

**Keywords:** air leak, chronic fistula, fistula, leak, occult bronchopleural fistula, persistent air leak

## Abstract

We present the case of a 67-year-old male patient who was brought to the ICU after cardiac arrest. Advanced cardiovascular life support (ACLS) measures, including chest compressions, resulted in bilateral pneumothoraces requiring bilateral chest tubes. A persistent air leak was noted on the right side, leading to a bronchopleural fistula, which required constant suction and significantly limited the treatment options that could be offered to the patient. He ultimately succumbed to his injuries because the degree of suction required to inflate his lungs was incompatible with transportation options.

## Introduction

Advanced cardiovascular life support (ACLS) and cardiopulmonary resuscitation (CPR) are critical interventions for managing cardiac arrest and other life-threatening conditions. While lifesaving, both procedures are associated with various complications. Commonly reported issues include rib fractures, sternal fractures, pneumothorax, hemothorax, injury to surrounding organs (e.g., liver and spleen), myocardial contusion, and bronchopleural fistula [1-2]. Our patient experienced several of these complications, the most significant being a severe bronchopleural fistula.

A bronchopleural fistula is defined as a pathological connection between the mainstem, lobar, or segmental bronchus and the pleural space. While most commonly resulting from lung resection surgery, the present case involves a trauma-induced bronchopleural fistula following ACLS measures, including chest compressions [3-4].

This report aims to highlight the clinical challenges associated with managing a chronic bronchopleural fistula as a complication of ACLS.

## Case presentation

This is the case of a 67-year-old male patient with a past medical history significant for schizophrenia and polysubstance use disorder who initially presented to Arrowhead Regional Medical Center (ARMC), Colton, CA, at the behavioral health unit after being arrested by police for driving erratically. He was being walked into the building when he became unresponsive just outside the main entrance. The patient was deemed to be in cardiac arrest; therefore, a code blue was announced, mechanical chest compressions were begun by police and then nursing staff of the behavioral health unit, emergency medicine physicians responded to the scene, and the patient was subsequently intubated. En route to the emergency room via gurney, the patient was manually ventilated via bag and was given 2 mg of epinephrine, 1 amp of bicarbonate, 2 doses of Narcan, and calcium. Return of spontaneous circulation was achieved.

**Figure 1:**
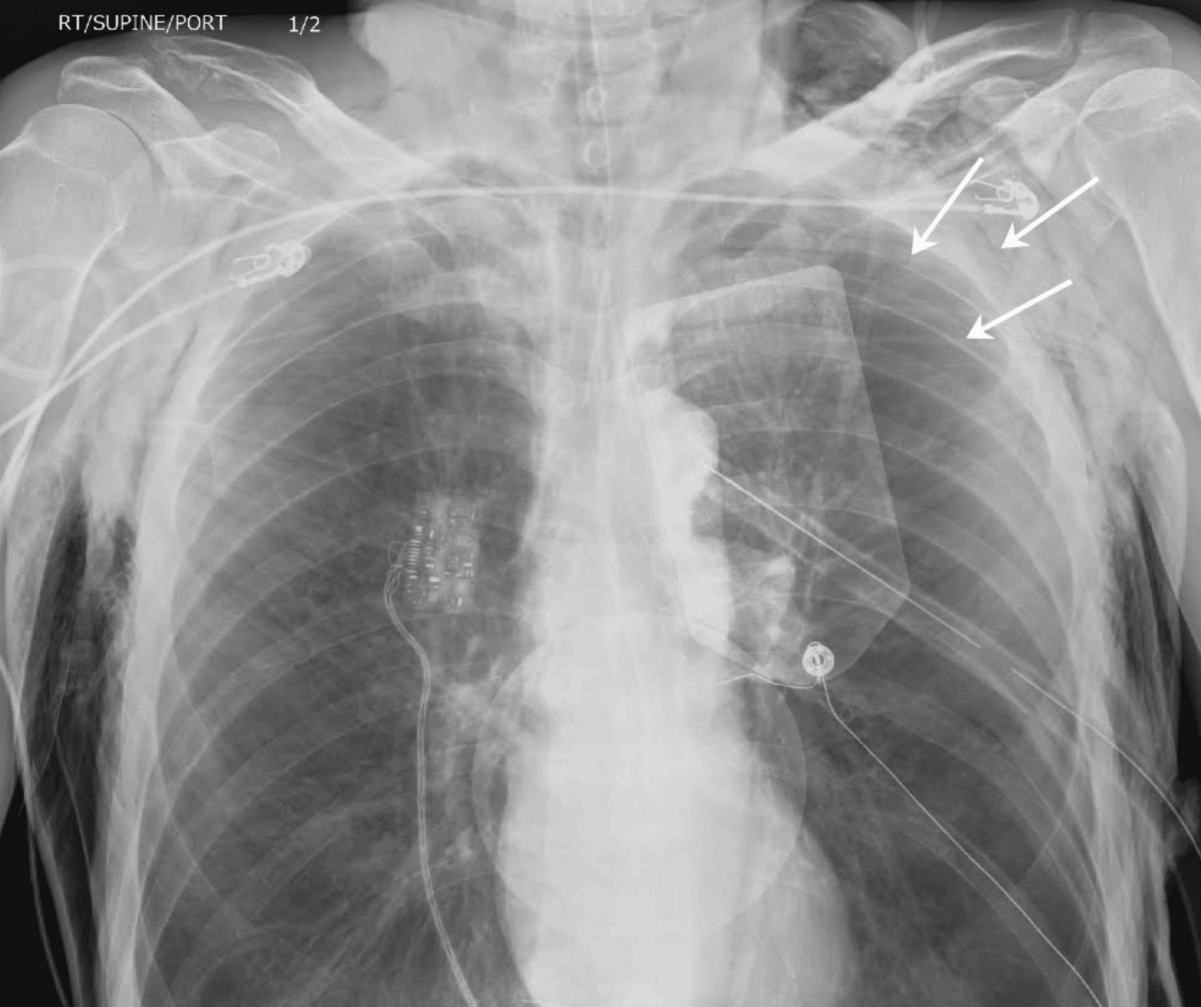
The chest X-ray demonstrates left pneumothorax (arrows) and lucency projecting over the left scapula suggestive of a non-displaced fracture. A chest tube and defibrillation pad can be seen on the left. Telemetry lead is seen on the right.

**Figure 2:**
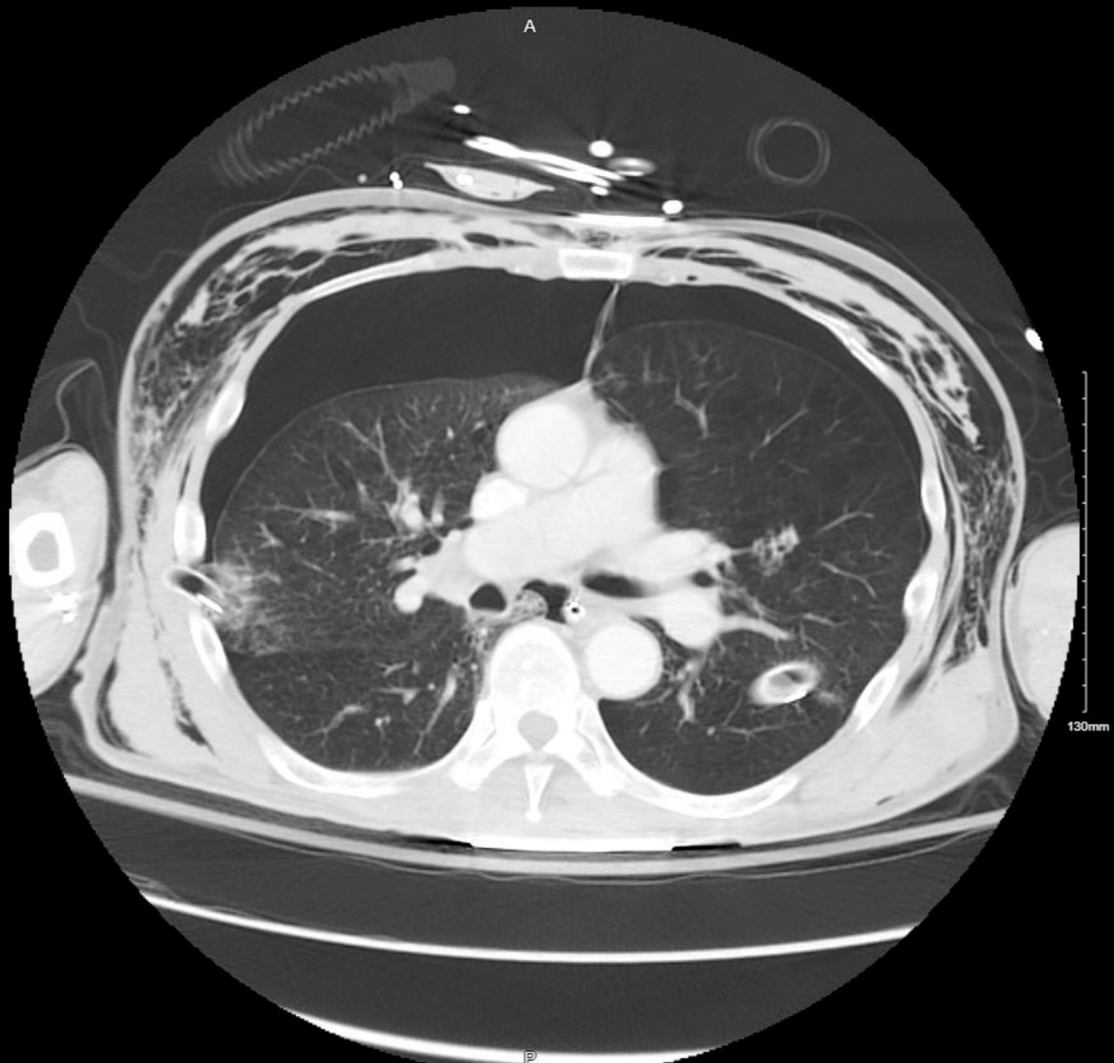
An initial CT scan of the chest demonstrates bilateral pneumothoraces. Of note, the left chest drain is potentially penetrating the left lung parenchyma.

Upon arrival at the hospital, an initial chest X-ray (CXR) demonstrated left pneumothorax, possible right pneumothorax, and sternal/rib fractures likely due to CPR. As a result, a left-sided chest tube was placed (Figure 1). A CT chest was then done, which confirmed the suspected bilateral pneumothoraces (Figure 2). His emergency department course was noteworthy for fentanyl/amphetamines in the urine drug screen, and blood cultures were later positive for *Haemophilus influenzae*, which was treated with intravenous ceftriaxone for seven days. He was ultimately intubated and sedated for airway protection.

**Figure 3:**
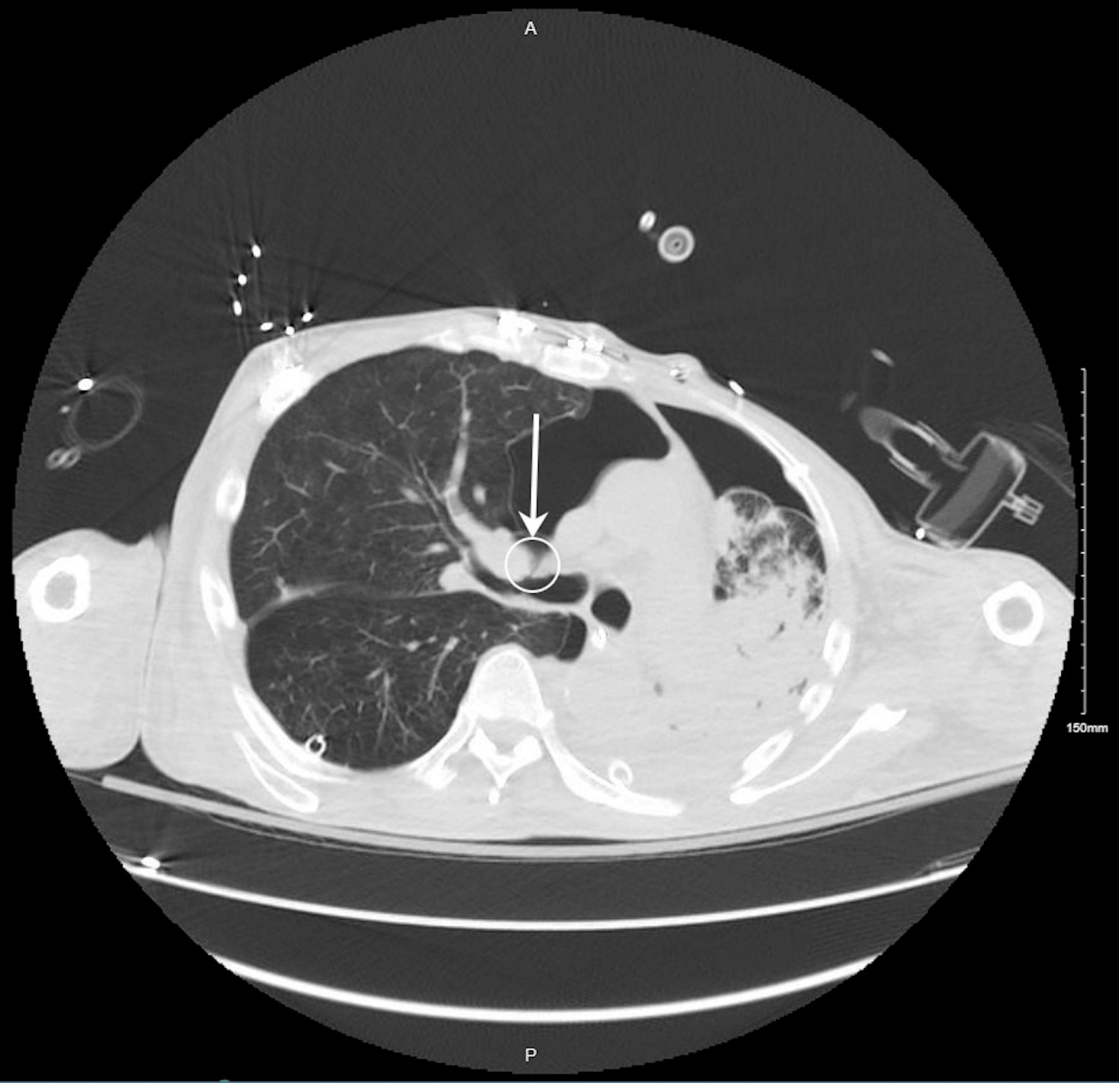
A repeat CT scan of the chest demonstrates a persistent large right and smaller left pneumothoraces with bilateral chest tubes in place. There is significant left lung consolidation/volume loss with left-shifting mediastinum. The broncho-pleural fistula is circled with arrows added.

While in the ICU, he developed worsening subcutaneous emphysema involving the neck. The general surgery team recommended and placed bilateral apical thoracic vents (a flexible catheter connected to a one-way valve and a self-sealing port), which direct air to escape the thoracic cavity and allow for re-expansion of the lung. The respiratory therapy team suspected an air leak; therefore, a CT scan of the chest was performed to identify the cause. This CT led to the discovery of possible penetration of the chest tube into the lung parenchyma (Figure 3). Cardiothoracic surgery was consulted and removed the right chest tube as well as the thoracic vent, replacing them with a pigtail catheter. “Blow-hole” incisions were created bilaterally for management of the subcutaneous emphysema.

A repeat CT scan of the chest was ordered due to persistent oxygen requirements and re-evaluation of the known pneumothoraces. It is at this point that a broncho-pleural fistula was first identified. The severity of the fistula was not yet known. Further into the hospital course, video-assisted thoracic surgery was performed by our cardiothoracic surgeon and revealed a right upper lobe bleb that was subsequently removed. At this point, it was unclear why the cardiothoracic surgery team did not address the known fistula. It is speculated that this management was being deferred to the pulmonology team, who later recruited the help of an interventional pulmonologist at the University of San Diego. 

**Figure 4:**
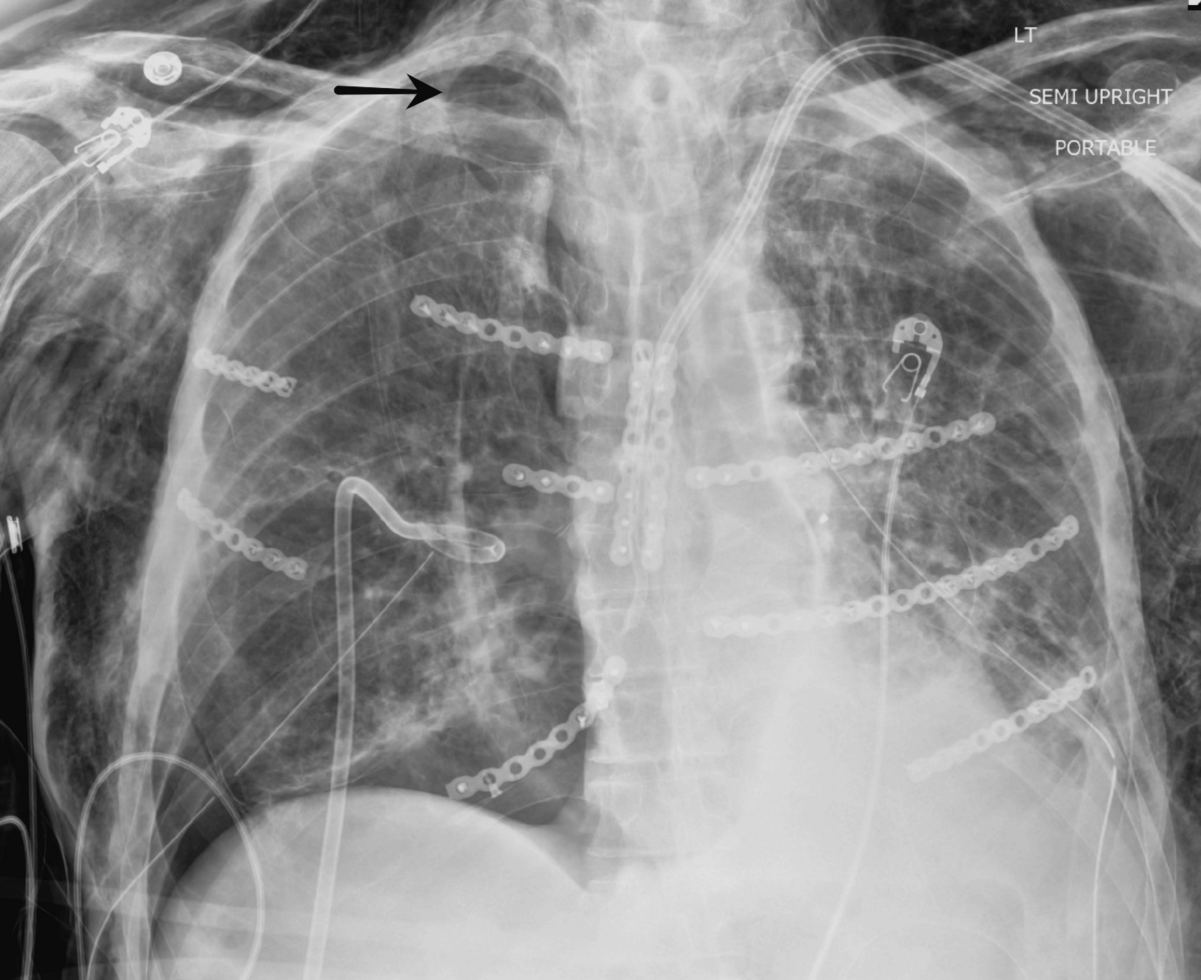
A chest X-ray with right apical pneumothorax (black arrow)

Tracheostomy was eventually performed due to the lengthy intubation period. Bilateral chest tubes remained in place with a significant suction force required to minimize intrapleural space air buildup. Later, an apical pneumothorax developed on the right side (Figure 4). This finding, in combination with a new loculated pneumothorax in the right lower lobe, was likely the cause of acute hemodynamic instability, thus requiring the placement of a third chest tube. 

Interventional pulmonology services were sought out at another facility to surgically repair the severe right-sided bronchopleural fistula. However, this proved challenging due to hemodynamic instability and the need for constant suction of the air leak. The patient was accepted by an interventional pulmonologist at the University of California, San Diego; however, concerns regarding transportation safety delayed transfer. Specifically, the amount of wall-mounted suction required to keep the lungs expanded was greater than what could be provided via portable suction devices. The ICU staff attempted to wean the patient off wall-mounted suction devices; however, this proved improbable as the patient would quickly desaturate below 80% SpO2. Similarly, when testing the option of portable suction devices, he would also desaturate below 80% SpO2. It is estimated that the patient required the maximum available negative pressure suction, which was approximately -600 mmHg. At this point in the hospitalization process, the patient's treatment options became limited to only what could be provided in his room while on the highest degree of negative pressure wall suction. Because of the suction requirements, our patient became bound to his hospital bed for several months while critical care staff attempted to wean him off wall suction without success. Multiple conversations were had with the patient and his family regarding his goals of care. On each occasion, the decision was made to pursue all viable medical options and keep his code status as full code. At no point did the patient or his family agree to hospice or palliative care options. 

**Figure 5:**
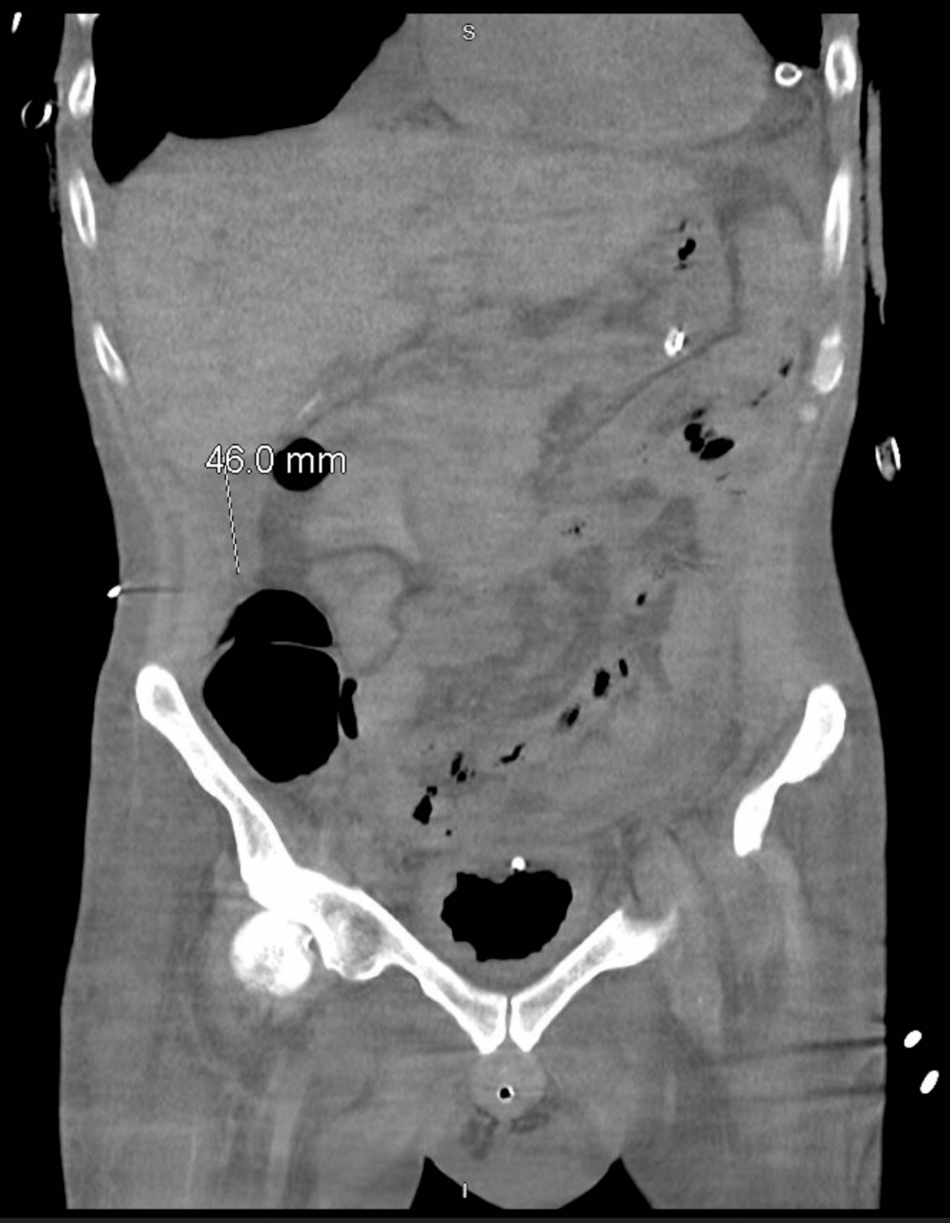
A CT scan of the abdomen demonstrates fluid collection below the liver which spans 46 mm.

After being hospitalized for three months, our patient began to complain of right upper quadrant pain. X-ray imaging of the chest, abdomen, and pelvis was unremarkable; therefore, a CT of the abdomen and pelvis was ordered. For this, he was placed on portable suction devices and quickly transported to the CT imaging suite. He was noted to desaturate during the process, but successfully made it to the imaging suite, where he could be placed back on wall suction. CT imaging of the abdomen and pelvis noted a subhepatic abscess (Figure 5). A decision was then made to have interventional radiology drain the abscess; however, it is unclear if his suction requirements were adequately described to the interventional radiology (IR) staff. It is also unclear for how long he was on portable suction devices instead of wall suction. Upon his arrival to the interventional radiology suite, pulses were lost, and ACLS protocol was initiated per code status at the time. Unfortunately, return of spontaneous circulation could not be achieved, and the patient passed away. 

## Discussion

Trauma causes may result in a bronchopleural fistula by creating a direct disruption of the tracheobronchial wall that communicates with the pleural space, with subsequent failure of the defect to seal due to ongoing pressure gradients, infection, or tissue devitalization. This patient was at high risk for both hospital-acquired pneumonia and tissue devitalization due to his older age and history of polysubstance use. The fistula was perpetuated by his need for positive pressure ventilation, which increases the driving pressure across the defect, sustaining flow through the fistula and preventing apposition of tissues [4].

Current literature emphasizes strategies to minimize airflow across the fistula, such as reducing peak inspiratory pressure, tidal volume, and positive end-expiratory pressure (PEEP). In refractory cases, lung isolation or independent lung ventilation may be considered [5]. Conservative management is appropriate for small, early-stage fistulas in stable patients, particularly in the context of spontaneous pneumothorax, where spontaneous closure may occur [6].

Endoscopic and bronchoscopic techniques are recommended for small- to intermediate-sized fistulas, high-risk surgical candidates, or as a bridge to definitive surgery. These interventions include the use of glues (cyanoacrylate, fibrin), coils, spigots, stents, silver nitrate, and expandable plugs. These techniques may be repeated and do not preclude subsequent surgical options. Endobronchial valves and occlusion devices are increasingly utilized, though long-term comparative data remain limited [7-8].

Surgical intervention is generally reserved for large, chronic, or refractory fistulas or when endoscopic treatments fail. Surgical options include direct repair of the bronchial stump, completion pneumonectomy, closure with muscle or omental flaps, and open window thoracostomy (OWT). The American Association for Thoracic Surgery recognizes OWT as a viable strategy for patients unfit for additional surgery or those with recurrent disease [9].

This case expands upon the existing literature by emphasizing the difficulty of managing chronic bronchopleural fistulas. Notably, the chronic (more than three months) nature of the fistula ultimately contributed to the patient’s death, suggesting that chronicity, rather than size alone, may be a key determinant in disease severity. Another major limitation to this patient's treatment plan was the need for constant wall suction. Current literature suggests that wall suction and portable suction devices typically provide equal suction capabilities (300 torr) [10]; however, variable effects of portable suction devices cannot be ruled out as a cause of this patient's sudden demise. 

## Conclusions

A major complicating factor in this case was the chronicity of the bronchopleural fistula requiring constant negative-pressure suction. The patient remained hospitalized for several months and likely required a higher level of care to access advanced pulmonary interventions such as endobronchial valves or stents. However, reliance on portable suction devices posed a continuous risk of recurrent pneumothorax. This case suggests that there is variable reliability of the portable electronic devices when transporting the patient within the hospital. When taking this patient to various imaging modalities, intra-hospital transport was associated with significant desaturation risk, likely because of the variable effectiveness of portable transportation suction devices. Attempting transfer to another facility using only portable suction devices would have likely resulted in severe hypoxia or death.
